# Agmatine Sulfate Is Associated with Altered Redox Status, Apoptosis-Associated Responses, and AKT/JNK Signalling in Ishikawa Endometrial Cancer Cells

**DOI:** 10.3390/life16091483

**Published:** 2026-09-05

**Authors:** Neziha Senem Arı, Ayşe Çakır Gündoğdu

**Affiliations:** Department of Histology and Embryology, Faculty of Medicine, Kütahya Health Sciences University, Evliya Çelebi Campus, Tavşanlı Road 10th km, Kütahya 43100, Türkiye

**Keywords:** agmatine sulfate, endometrial cancer, oxidative stress, apoptosis, AKT/JNK signalling

## Abstract

Agmatine is a naturally occurring polyamine with context-dependent effects on cellular redox regulation, survival, and apoptosis; however, its effects in endometrial cancer cells remain poorly characterized. This exploratory *in vitro* study investigated cellular responses associated with agmatine sulfate exposure in Ishikawa endometrial adenocarcinoma cells, using human dermal fibroblasts (HDFs) as a non-cancerous, non-tissue-matched comparator. Metabolic activity was assessed using the 3-(4,5-dimethylthiazol-2-yl)-2,5-diphenyltetrazolium bromide (MTT) assay following exposure to 0.5–15 mM agmatine sulfate for 24 and 48 h. Based on the 48-h concentration-response analysis in Ishikawa cells, 4.1 and 6.5 mM were selected for subsequent analyses of intracellular reactive oxygen species (ROS), malondialdehyde (MDA), cleaved caspase-3, and p-AKT/p-JNK immunoreactivity. Agmatine sulfate produced concentration- and time-dependent reductions in MTT-derived metabolic activity in both cell lines. In Ishikawa cells, 4.1 and 6.5 mM agmatine sulfate significantly increased ROS, MDA, and cleaved caspase-3 levels, accompanied by decreased p-AKT and increased p-JNK immunoreactivity. In HDF cells, ROS levels increased at both concentrations, whereas significant increases in MDA, cleaved caspase-3, and p-JNK were observed only at 6.5 mM; p-AKT immunoreactivity remained unchanged. These findings indicate that agmatine sulfate exposure is associated with reduced metabolic activity, increased oxidative stress and apoptosis-associated responses, and changes in AKT/JNK-related signalling in Ishikawa cells. However, the responses observed in HDF cells do not support a conclusion of tumour-selective activity. Further studies using additional endometrial cancer cell lines, tissue-matched non-malignant controls, and targeted experimental approaches are warranted to clarify the biological relationships among these responses.

## 1. Introduction

Endometrial cancer is one of the most common gynaecological malignancies and is characterized by considerable molecular and biological heterogeneity [1]. Among the signalling networks involved in endometrial carcinogenesis, the phosphatidylinositol 3-kinase/protein kinase B (PI3K/AKT) pathway regulates cellular growth and survival. Aberrant activation of the PI3K/AKT/mechanistic target of rapamycin (mTOR) pathway has been implicated in the pathogenesis of endometrial cancer [2]. In parallel, c-Jun N-terminal kinases (JNKs), members of the mitogen-activated protein kinase (MAPK) family, mediate cellular responses to a wide range of extracellular and intracellular stress stimuli [3]. Cross-talk between survival-associated PI3K/AKT signalling and stress-responsive JNK signalling may therefore influence cellular responses to stress and cell-death stimuli [4].

Oxidative stress is a well-described feature of cancer biology. Cancer cells commonly exhibit elevated reactive oxygen species (ROS) levels and altered redox homeostasis. Moderately elevated ROS levels may support tumour-cell growth and oncogenic signalling, whereas excessive ROS accumulation can become cytotoxic when cellular antioxidant capacity is exceeded, damaging cellular macromolecules, promoting lipid peroxidation, and activating stress-responsive signalling and cell-death mechanisms [5,6]. Malondialdehyde (MDA), a major product of polyunsaturated fatty acid peroxidation, is widely used as a biomarker of lipid peroxidation and oxidative stress [7]. Oxidative stress can trigger intrinsic apoptotic signalling, which ultimately converges on the activation of effector caspases, including caspase-3 [8]. Accordingly, the combined assessment of ROS generation, lipid peroxidation, and cleaved caspase-3 describes both the oxidative burden and the apoptotic response of the cell [9]. The dual role of ROS is particularly relevant to endometrial cancer, in which ROS-mediated redox alterations can modulate multiple signalling pathways, including PI3K/AKT and MAPK signalling [10].

Agmatine is a naturally occurring polyamine derived from L-arginine via arginine decarboxylase and has been implicated in a broad range of physiological and pathological processes, including the regulation of cellular function and cytoprotection [11]. The cellular effects of agmatine reported to date are inconsistent across experimental systems, ranging from cytoprotective and antioxidant actions in some non-malignant models to antiproliferative and pro-apoptotic effects in certain tumour-cell models, suggesting that its biological effects may depend on the cell type and context examined. Several studies have demonstrated antioxidant and cytoprotective effects of agmatine in non-malignant experimental injury models [12,13]. For example, agmatine has been reported to suppress oxidative stress through PI3K/AKT-dependent activation of Nrf2/HO-1 signalling in macrophages [12] and to exert cytoprotective effects against cisplatin-induced ototoxicity through activation of PI3K/AKT signalling [13]. Whether this cytoprotection extends to malignant cells is less clear.

Evidence from tumour-cell models suggests that agmatine may exert biological effects distinct from those observed in non-malignant injury models. In Caco-2 colorectal adenocarcinoma cells, agmatine has been reported to exert antiproliferative, anti-migratory, anti-invasive, and pro-apoptotic effects [14]. Agmatine sulfate has also been reported to decrease PI3K and Akt-1 expression levels in HT1080 human fibrosarcoma cells in association with modulation of MMP-2 [15]. This contrasts with the activation of PI3K/AKT signalling reported in several non-malignant cytoprotective models [12,13], further suggesting that the signalling responses to agmatine may depend on the cellular and experimental context.

JNK is of particular interest in this context because oxidative and other cellular stresses can modulate JNK signalling, which may participate in either cell-survival or cell-death responses depending on the cellular context [3]. Accordingly, p-AKT and p-JNK were measured together to describe the signalling responses accompanying agmatine exposure.

Despite growing interest in agmatine, its effects in endometrial cancer cells remain poorly characterized. In particular, limited information is available regarding the effects of agmatine on metabolic activity, oxidative stress, apoptosis-associated responses, and AKT/JNK signalling in endometrial cancer cells. Therefore, the present exploratory *in vitro* study investigated the concentration-dependent effects of agmatine sulfate on MTT-derived metabolic activity in Ishikawa human endometrial adenocarcinoma cells and human dermal fibroblasts (HDFs). Based on the concentration-response analysis, selected concentrations were subsequently used to assess intracellular ROS, MDA, and cleaved caspase-3, as well as p-AKT and p-JNK immunoreactivity. HDF cells were included as a non-cancerous, non-tissue-matched comparator to provide information on cell-context-dependent responses rather than as a surrogate for normal endometrial tissue. The study was designed to characterize cellular response patterns associated with agmatine sulfate exposure rather than to establish anticancer efficacy or causal regulation of AKT/JNK signalling.

## 2. Materials and Methods

**Figure 1:**
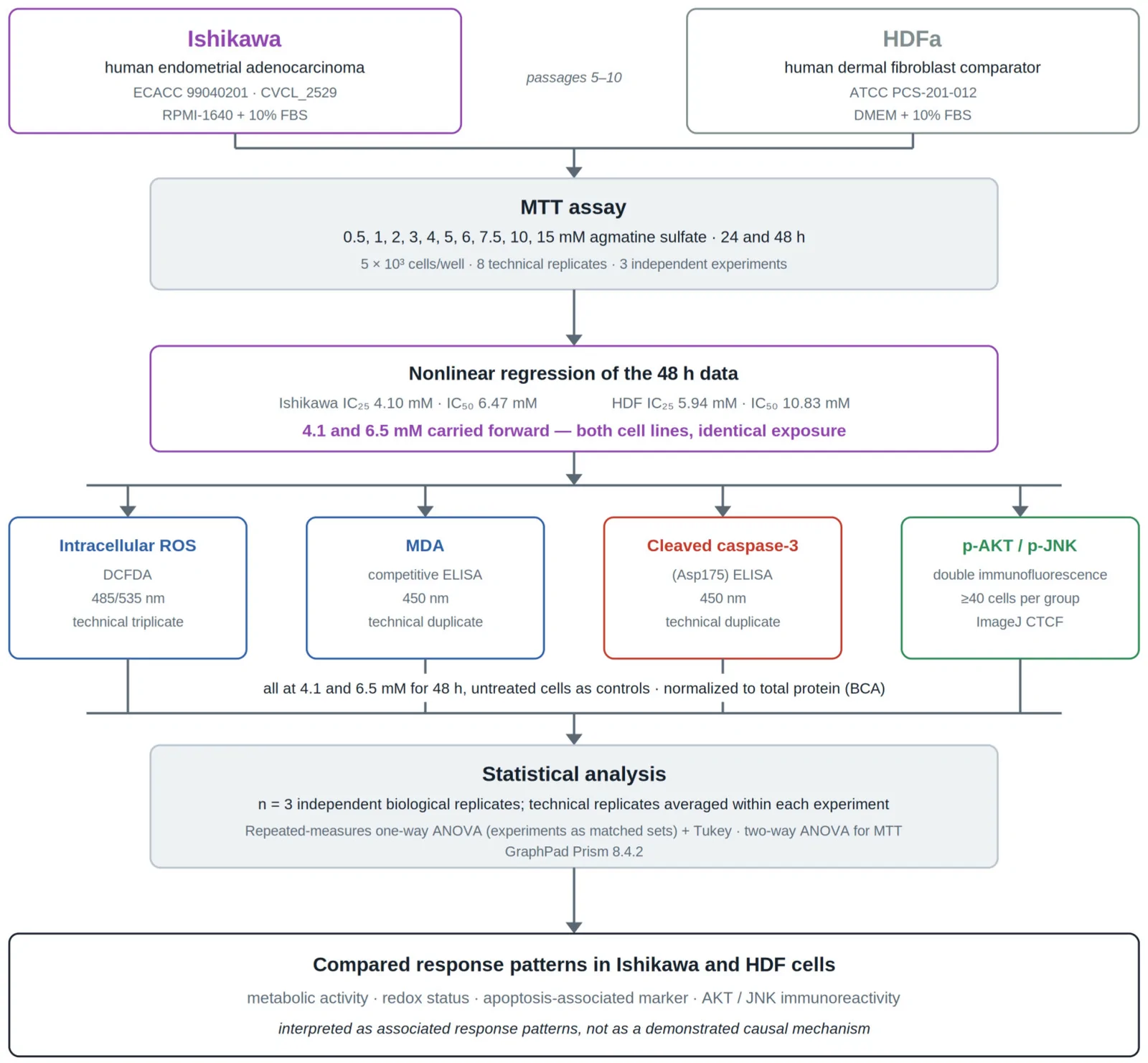
Overview of the experimental workflow. Ishikawa endometrial adenocarcinoma cells and HDFa dermal fibroblasts were exposed to 0.5–15 mM agmatine sulfate for 24 and 48 h, and MTT-derived metabolic activity was measured. IC_25_ and IC_50_ values were estimated by nonlinear regression of the 48-h data, and the Ishikawa IC_25_ (4.1 mM) and IC_50_ (6.5 mM) were applied to both cell lines in all downstream assays under identical exposure conditions. Intracellular ROS, MDA, cleaved caspase-3 (Asp175), and p-AKT/p-JNK immunoreactivity were then assessed after 48 h, with untreated cells as controls and all biochemical readouts normalized to total protein content. Each experiment was performed three times independently (*n* = 3 independent biological replicates). BCA, bicinchoninic acid; CTCF, corrected total cell fluorescence; DCFDA, 2′,7′-dichlorofluorescein diacetate; HDF, human dermal fibroblast; MDA, malondialdehyde.

An overview of the experimental workflow is provided in Figure 1.

### 2.1. Cell Lines and Culture Conditions

The Ishikawa human endometrial adenocarcinoma cell line used in the present study originated from the European Collection of Authenticated Cell Cultures (ECACC; Salisbury, UK; catalogue no. 99040201) and is registered in Cellosaurus under accession no. CVCL_2529. Human dermal fibroblasts (HDFa; ATCC PCS-201-012™; American Type Culture Collection, Manassas, VA, USA) were used as a non-cancerous, non-tissue-matched comparator rather than as a normal endometrial control.

Ishikawa cells were cultured in RPMI-1640 medium (Gibco; Thermo Fisher Scientific, Inc., Waltham, MA, USA), whereas HDFa cells were cultured in DMEM (Gibco; Thermo Fisher Scientific, Inc., Waltham, MA, USA). Both media were supplemented with 10% fetal bovine serum (FBS; Gibco, Cat. no. 10270-106, Grand Island, NY, USA), 1% L-glutamine (Gibco, Cat. no. 25030-024, Grand Island, NY, USA), 100 U/mL penicillin, and 0.1 mg/mL streptomycin (Gibco, Cat. no. 15140-122, Grand Island, NY, USA). Cells were maintained at 37 °C in a humidified atmosphere containing 5% CO_2_ and were used between passages 5 and 10. At approximately 80–90% confluence, cells were detached using 0.25% trypsin–EDTA (Gibco, Cat. no. 25200-056, Grand Island, NY, USA), counted, and seeded into the appropriate culture plates for subsequent experiments.

### 2.2. Assessment of Cellular Metabolic Activity by MTT Assay

The effect of agmatine sulfate on cellular metabolic activity was evaluated using the MTT [3-(4,5-dimethylthiazol-2-yl)-2,5-diphenyltetrazolium bromide] colorimetric assay. Briefly, Ishikawa and HDF cells were seeded into 96-well plates (Corning, Cat. no. 3596, Corning, NY, USA) at a density of 5 × 10^3^ cells/well and allowed to adhere overnight. Cells were subsequently treated with agmatine sulfate (Thermo Scientific, Waltham, MA, USA; Cat. no. H55363.03; CAS no. 2482-00-0; purity 97%) at 0.5, 1, 2, 3, 4, 5, 6, 7.5, 10, and 15 mM for 24 or 48 h. The concentration range was selected to encompass the millimolar concentrations previously used to investigate the cellular effects of agmatine in tumour-cell models [14,16,17]. Fresh working solutions of agmatine sulfate were prepared immediately before each experiment by dissolving the compound directly in the complete culture medium corresponding to each cell line. No organic solvent was used during solution preparation or dilution.

Following treatment, 10 μL of MTT solution (5 mg/mL; Elabscience, Houston, TX, USA; Cat. no. E-CK-A341) was added to each well containing 100 μL of culture medium, and the plates were incubated for 4 h at 37 °C. The culture medium was then carefully aspirated, and the resulting formazan crystals were dissolved in 100 μL of dimethyl sulfoxide (DMSO; Sigma-Aldrich, Cat. no. D2650, St. Louis, MO, USA). After gentle shaking to ensure complete dissolution of the crystals, absorbance was measured at 570 nm using a Multiskan™ FC Microplate Photometer (Thermo Fisher Scientific, Inc., Waltham, MA, USA).

The experiment was independently performed three times. Within each independent experiment, each treatment condition was analysed in eight technical replicate wells. The mean of the eight technical replicates from each independent experiment was used as the value for that experiment, and the three independent experiments were used for statistical analysis (*n* = 3 independent biological replicates).

MTT-derived metabolic activity was expressed as a percentage of the untreated control. Concentration-response curves were generated from the 48-h MTT data using nonlinear regression analysis, and the IC_25_ and IC_50_ values were determined separately for each cell line. Based on the 48-h concentration-response analysis in Ishikawa cells, 4.1 and 6.5 mM agmatine sulfate were selected as the approximate IC_25_ and IC_50_ concentrations, respectively. These same concentrations were subsequently applied to both Ishikawa and HDF cells in the downstream assays to enable direct comparison of treatment responses under identical exposure conditions.

### 2.3. Intracellular ROS Measurement

Intracellular ROS levels were assessed using the DCFDA/H_2_DCFDA Cellular ROS Assay Kit (Abcam, Cat. no. ab113851, Cambridge, UK) according to the manufacturer’s guidelines, with minor adjustments for adherent cell cultures. Ishikawa and HDF cells were seeded into black-walled 96-well plates (Corning, Cat. no. 3603, Corning, NY, USA) and allowed to adhere overnight. Cells were then treated with 4.1 or 6.5 mM agmatine sulfate for 48 h, while untreated cells served as controls. The tert-butyl hydroperoxide (TBHP) positive control supplied with the kit was used according to the manufacturer’s instructions.

Following treatment, the wells were washed once with the assay buffer supplied with the kit and incubated with 20–25 μM DCFDA in the same buffer for 30 min at 37 °C in the dark. After dye loading, the wells were washed again with the assay buffer, and fluorescence was measured using a Varioskan LUX multimode microplate reader (Thermo Fisher Scientific, Inc., Waltham, MA, USA) at excitation/emission wavelengths of 485/535 nm. Background fluorescence from blank wells was subtracted, and ROS-associated fluorescence was normalized to the total protein content per well, as determined using a bicinchoninic acid (BCA) protein assay (Pierce™ BCA Protein Assay Kit, Thermo Scientific, Cat. no. 23225, Waltham, MA, USA). Protein-normalized fluorescence values were subsequently expressed relative to the untreated control, which was set to 1, and reported as fold change.

Each condition was analysed in technical triplicate, and the experiment was independently repeated three times (*n* = 3 independent biological replicates). The mean of the technical replicates from each independent experiment was used for statistical analysis.

### 2.4. Malondialdehyde (MDA) Measurement

Malondialdehyde (MDA) levels were determined using a commercial competitive ELISA kit (Elabscience, Houston, TX, USA; Cat. no. E-EL-0060) according to the manufacturer’s instructions. Ishikawa and HDF cells were treated for 48 h with agmatine sulfate at 4.1 or 6.5 mM, while untreated cells served as controls.

Following treatment, cells were washed twice with ice-cold phosphate-buffered saline (PBS; Gibco, Cat. no. 10010-023, Grand Island, NY, USA), harvested, and resuspended in ice-cold PBS. Samples were maintained on ice during preparation and subsequently centrifuged. The resulting supernatants were collected and used for MDA determination. A 50 μL aliquot of each sample was added to the ELISA plate, and absorbance was measured at 450 nm using a Multiskan™ FC Microplate Photometer (Thermo Fisher Scientific, Inc., Waltham, MA, USA).

MDA concentrations were calculated from the standard curve and normalized to the total protein content of the corresponding samples, as determined using a bicinchoninic acid (BCA) protein assay. Normalized MDA levels were expressed as ng/mg protein.

Each experimental condition was analysed in technical duplicate, and the experiment was independently repeated three times (*n* = 3 independent biological replicates). The mean of the technical duplicates from each independent experiment was used for statistical analysis.

### 2.5. Cleaved Caspase-3 (Asp175) Measurement

Cleaved caspase-3 (Asp175) levels were quantified using the Human Cleaved Caspase-3 (Asp175) SimpleStep ELISA^®^ Kit (ab220655, Abcam, Cambridge, UK) according to the manufacturer’s instructions. Ishikawa and HDF cells were treated for 48 h with agmatine sulfate at 4.1 or 6.5 mM, while untreated cells served as controls.

Following treatment, cells were washed twice with ice-cold phosphate-buffered saline (PBS) and lysed using chilled 1× Cell Extraction Buffer PTR supplied with the kit. Samples were maintained on ice during extraction and subsequently centrifuged. The resulting supernatants were collected and used for cleaved caspase-3 determination. A 50 μL aliquot of each sample was added to the ELISA plate, and absorbance was measured at 450 nm using a Multiskan™ FC Microplate Photometer (Thermo Fisher Scientific, Inc., Waltham, MA, USA).

Cleaved caspase-3 concentrations were calculated from the standard curve and normalized to the total protein content of the corresponding samples, as determined using a bicinchoninic acid (BCA) protein assay. Normalized cleaved caspase-3 levels were expressed as pg/mg protein.

For the cleaved caspase-3 assay, each experimental condition was analysed in technical duplicate, and the experiment was independently repeated three times (*n* = 3 independent biological replicates). The mean of the technical duplicates from each independent experiment was used for statistical analysis.

### 2.6. Immunofluorescence Staining for p-JNK and p-AKT

Phosphorylated c-Jun N-terminal kinase (p-JNK) and phosphorylated AKT (p-AKT) immunoreactivity were evaluated by double immunofluorescence staining. Ishikawa and HDF cells were seeded onto sterile 13-mm glass coverslips (VWR, Cat. no. 631-0150, Darmstadt, Germany) in 24-well plates (Corning, Cat. no. 3524, Corning, NY, USA) and treated with 4.1 or 6.5 mM agmatine sulfate for 48 h, while untreated cells served as controls. Cells were fixed with 4% paraformaldehyde (Sigma-Aldrich, Cat. no. P6148, St. Louis, MO, USA) for 20 min, permeabilized with 0.1% Triton X-100 (Sigma-Aldrich, Cat. no. T8787, St. Louis, MO, USA) for 15 min, and blocked with 3% bovine serum albumin (BSA; Sigma-Aldrich, Cat. no. A9647, St. Louis, MO, USA) for 30 min at room temperature.

Cells were incubated overnight at 4 °C with rabbit polyclonal anti-AKT1 (phospho-Ser473) antibody (Abcam, Cambridge, UK; Cat. no. ab8932; 1:100) and mouse monoclonal anti-phospho-JNK1/JNK2 (Thr183/Tyr185) antibody (Invitrogen, Carlsbad, CA, USA, Cat. no. MA5-15228; clone E.665.10; 1:200). Following washing with PBS, cells were incubated for 1 h at room temperature with Alexa Fluor^®^ 594-conjugated goat anti-rabbit IgG (Jackson ImmunoResearch, Cat. no. 111-585-003, West Grove, PA, USA; 1:200) and Alexa Fluor^®^ 488-conjugated goat anti-mouse IgG (Jackson ImmunoResearch, Cat. no. 115-545-003, West Grove, PA, USA; 1:200). Nuclei were counterstained and coverslips were mounted using Fluoroshield™ with DAPI (Sigma-Aldrich, Cat. no. F6057, St. Louis, MO, USA). 

Fluorescence images were acquired using a Zeiss fluorescence microscope (Carl Zeiss Microscopy, Oberkochen, Germany) under identical acquisition settings across treatment groups within each cell line. The DAPI, Alexa Fluor 488 (p-JNK), and Alexa Fluor 594 (p-AKT) channels were acquired separately and merged for visualization. Fluorescence intensity was quantified separately for p-JNK and p-AKT using ImageJ software (version 1.54, National Institutes of Health, Bethesda, MD, USA). Corrected total cell fluorescence (CTCF) was calculated as:

CTCF = Integrated density − (Cell area × Mean background fluorescence).

In each independent experiment, at least 40 cells per treatment group were analysed from randomly selected microscopic fields by an examiner blinded to treatment allocation. Three independent experiments were performed. For each marker and treatment condition, the cellular CTCF measurements within each independent experiment were averaged to obtain a single value per experiment. These independent experimental means were subsequently used for statistical analysis (*n* = 3 independent biological replicates).

### 2.7. Statistical Analysis

Statistical analyses were performed using GraphPad Prism version 8.4.2 (GraphPad Software, San Diego, CA, USA). Data are presented as mean ± standard deviation (SD) of three independent biological experiments (*n* = 3). For the MTT assay, the effects of agmatine sulfate concentration and treatment duration were analysed using ordinary two-way ANOVA followed by Dunnett’s multiple comparisons test, with each concentration compared with the untreated control at the corresponding time point. For ROS, MDA, cleaved caspase-3, and immunofluorescence analyses, treatment groups were analysed separately for each cell line using repeated-measures one-way ANOVA, with the three independent experiments entered as matched sets, followed by Tukey’s multiple comparisons test. IC_25_ and IC_50_ values were estimated from the 48-h MTT concentration-response data using nonlinear regression analysis. A *p* value < 0.05 was considered statistically significant. A prospective power calculation was not performed because this was an exploratory study and prior effect-size estimates for these specific endpoints in agmatine-treated Ishikawa cells were not available. Three independent biological replicates were used consistently across assays to provide independently repeated measurements of the observed responses.

## 3. Results

### 3.1. Effects of Agmatine Sulfate on MTT-Derived Metabolic Activity

**Figure 2:**
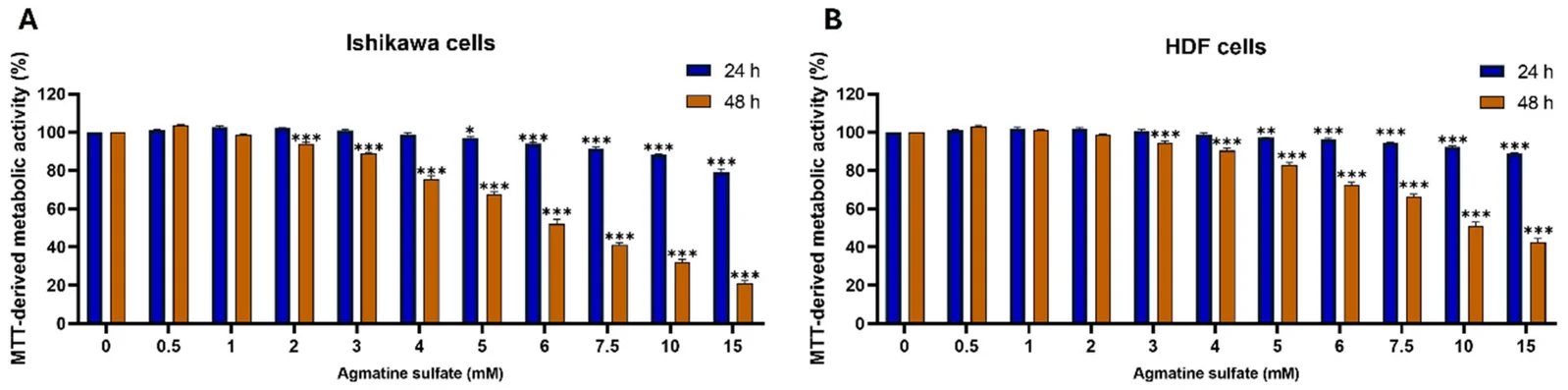
Effects of agmatine sulfate on MTT-derived metabolic activity in Ishikawa and HDF cells. (**A**) Ishikawa cells and (**B**) HDF cells were treated with increasing concentrations of agmatine sulfate (0–15 mM) for 24 or 48 h. MTT-derived metabolic activity was expressed as a percentage of untreated control cells. Data are presented as the mean ± SD of three independent experiments. Statistical significance was determined by ordinary two-way ANOVA followed by Dunnett’s multiple comparisons test, with each concentration compared with the untreated control at the corresponding time point. * *p* < 0.05, ** *p* < 0.01, *** *p* < 0.001.

Agmatine sulfate induced concentration- and time-dependent changes in MTT-derived metabolic activity in both Ishikawa and HDF cells (Figure 2A,B). In Ishikawa cells (Figure 2A), exposure to agmatine sulfate for 24 h resulted in only modest changes at lower concentrations, whereas significant reductions in MTT-derived metabolic activity were observed from 5 mM onwards. Following 48 h of treatment, the inhibitory effect became more pronounced, with significant decreases detected from 2 mM, indicating a clear concentration-dependent reduction in cellular metabolic activity.

Similarly, HDF cells (Figure 2B) exhibited concentration-dependent reductions in MTT-derived metabolic activity following agmatine sulfate treatment. At 24 h, significant decreases were observed from 5 mM onwards, whereas after 48 h, significant reductions were detected from 3 mM.

Ordinary two-way ANOVA confirmed significant effects of agmatine sulfate concentration, treatment duration, and their interaction (all *p* < 0.0001), supporting the observed concentration- and time-dependent reduction in MTT-derived metabolic activity.

Consistent with the MTT findings, nonlinear regression analysis of the 48-h concentration-response data yielded lower IC_25_ and IC_50_ values for Ishikawa cells than for HDF cells. The calculated IC_25_ and IC_50_ values were 4.10 and 6.47 mM for Ishikawa cells and 5.94 and 10.83 mM for HDF cells, respectively. Based on these findings, concentrations corresponding to the Ishikawa IC_25_ (4.1 mM) and IC_50_ (6.5 mM) were selected for all subsequent biochemical and immunofluorescence experiments in both cell lines to enable direct comparison of treatment responses under identical exposure conditions.

### 3.2. Effects of Agmatine Sulfate on Intracellular ROS Levels

**Figure 3:**
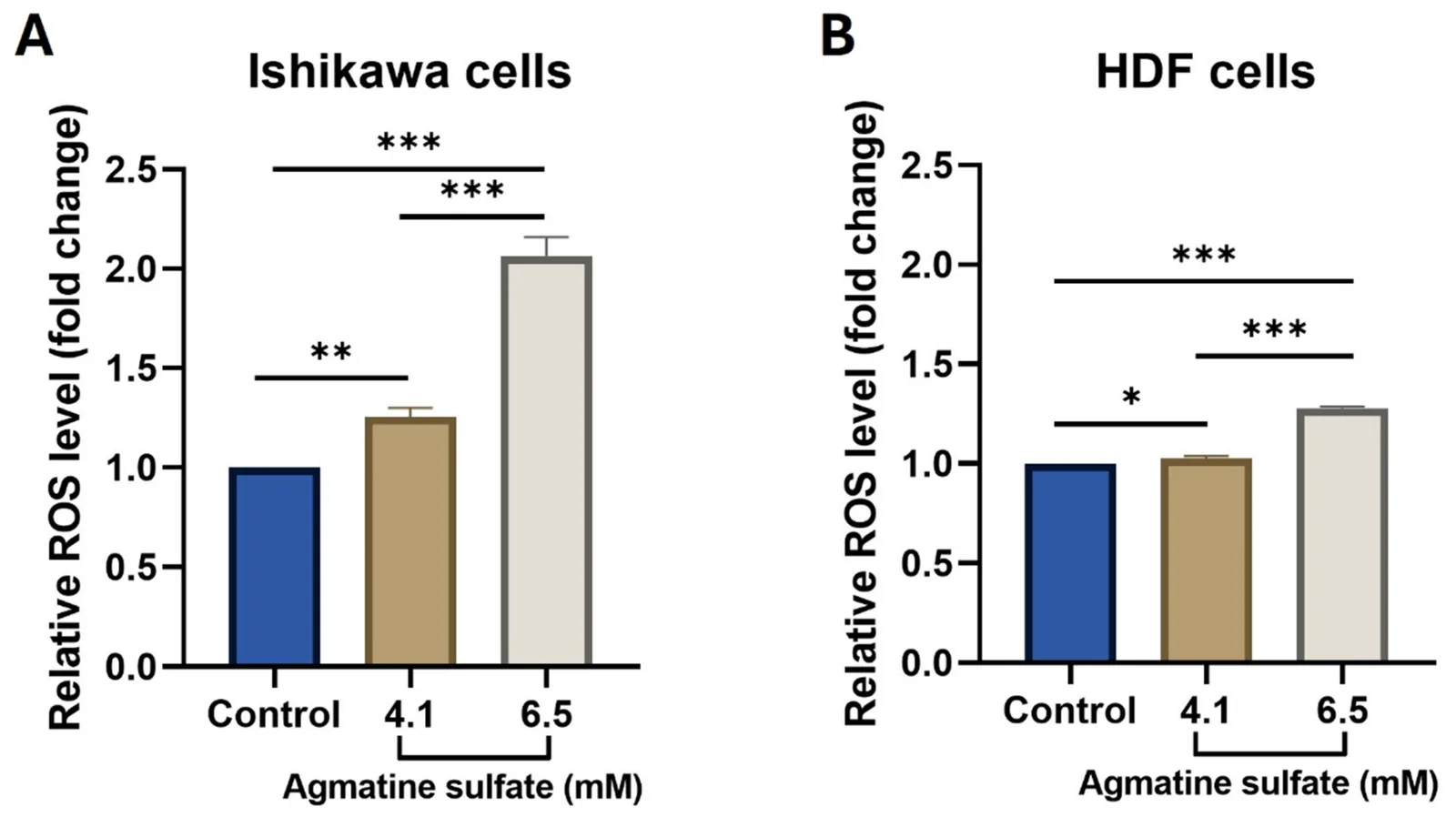
Effects of agmatine sulfate on intracellular ROS levels in Ishikawa and HDF cells. Relative intracellular ROS levels in (**A**) Ishikawa and (**B**) HDF cells following treatment with 4.1 or 6.5 mM agmatine sulfate for 48 h. Fluorescence values were normalized to total protein content and subsequently expressed relative to the untreated control, which was set to 1 for display purposes; statistical analysis was performed on the protein-normalized values before this scaling. Data are presented as mean ± SD of three independent experiments (*n* = 3 independent biological replicates). Statistical significance was determined separately for each cell line using repeated-measures one-way ANOVA followed by Tukey’s multiple comparisons test. Only statistically significant comparisons are indicated. * *p* < 0.05, ** *p* < 0.01, *** *p* < 0.001.

Agmatine sulfate significantly increased intracellular ROS levels in both Ishikawa and HDF cells following 48 h of treatment (Figure 3A,B). In Ishikawa cells (Figure 3A), relative ROS levels were significantly increased at 4.1 mM compared with the untreated control (*p* = 0.0059), with a more pronounced increase observed at 6.5 mM (*p* < 0.0001). Relative ROS levels were also significantly higher at 6.5 mM than at 4.1 mM (*p* < 0.0001).

In HDF cells (Figure 3B), treatment with 4.1 mM agmatine sulfate resulted in a modest but significant increase in relative ROS levels compared with the untreated control (*p* = 0.0249), whereas a greater increase was observed at 6.5 mM (*p* < 0.0001). Relative ROS levels at 6.5 mM were also significantly higher than those at 4.1 mM (*p* < 0.0001). Overall, relative ROS levels increased with increasing agmatine sulfate concentration in both cell lines. 

### 3.3. Effects of Agmatine Sulfate on MDA Levels

**Figure 4:**
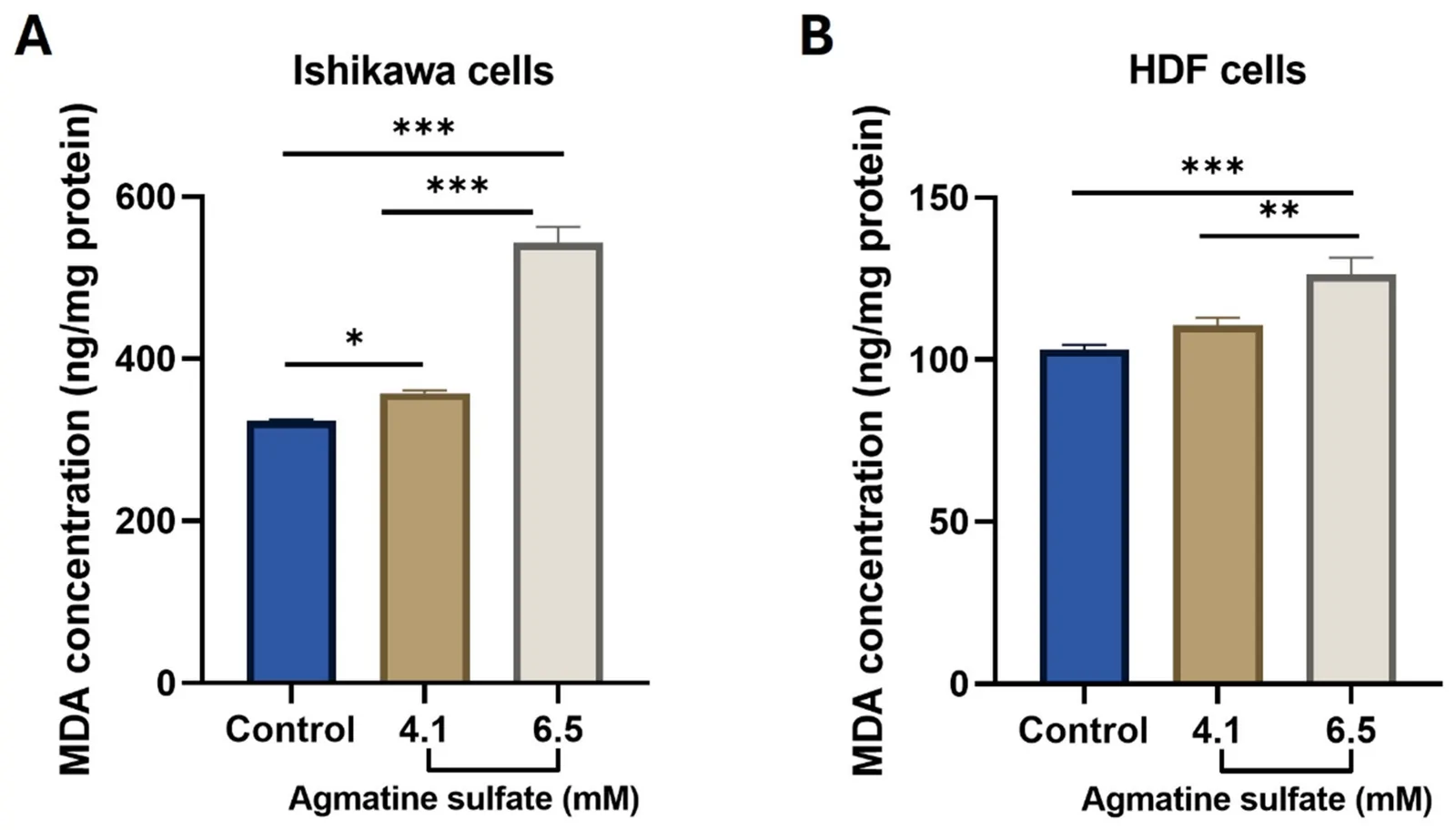
Effects of agmatine sulfate on MDA levels in Ishikawa and HDF cells. MDA levels in (**A**) Ishikawa and (**B**) HDF cells following treatment with 4.1 or 6.5 mM agmatine sulfate for 48 h. MDA levels were normalized to total protein content and expressed as ng/mg protein. Data are presented as mean ± SD of three independent experiments (*n* = 3). Statistical significance was determined separately for each cell line using repeated-measures one-way ANOVA followed by Tukey’s multiple comparisons test. Only statistically significant comparisons are indicated. * *p* < 0.05, ** *p* < 0.01, *** *p* < 0.001.

Agmatine sulfate increased MDA levels in both Ishikawa and HDF cells following 48 h of treatment (Figure 4A,B). In Ishikawa cells (Figure 4A), normalized MDA levels were significantly increased at 4.1 mM compared with the untreated control (*p* = 0.0239), with a substantially greater increase observed at 6.5 mM (*p* < 0.0001). MDA levels at 6.5 mM were also significantly higher than those at 4.1 mM (*p* < 0.0001).

In HDF cells (Figure 4B), treatment with 4.1 mM agmatine sulfate resulted in a modest increase in MDA levels that did not reach statistical significance compared with the untreated control (*p* = 0.0830). In contrast, MDA levels were significantly increased at 6.5 mM compared with both the untreated control (*p* = 0.0004) and the 4.1 mM treatment group (*p* = 0.0036). Overall, MDA levels increased with increasing agmatine sulfate concentration in both cell lines.

### 3.4. Effects of Agmatine Sulfate on Cleaved Caspase-3 Levels

**Figure 5:**
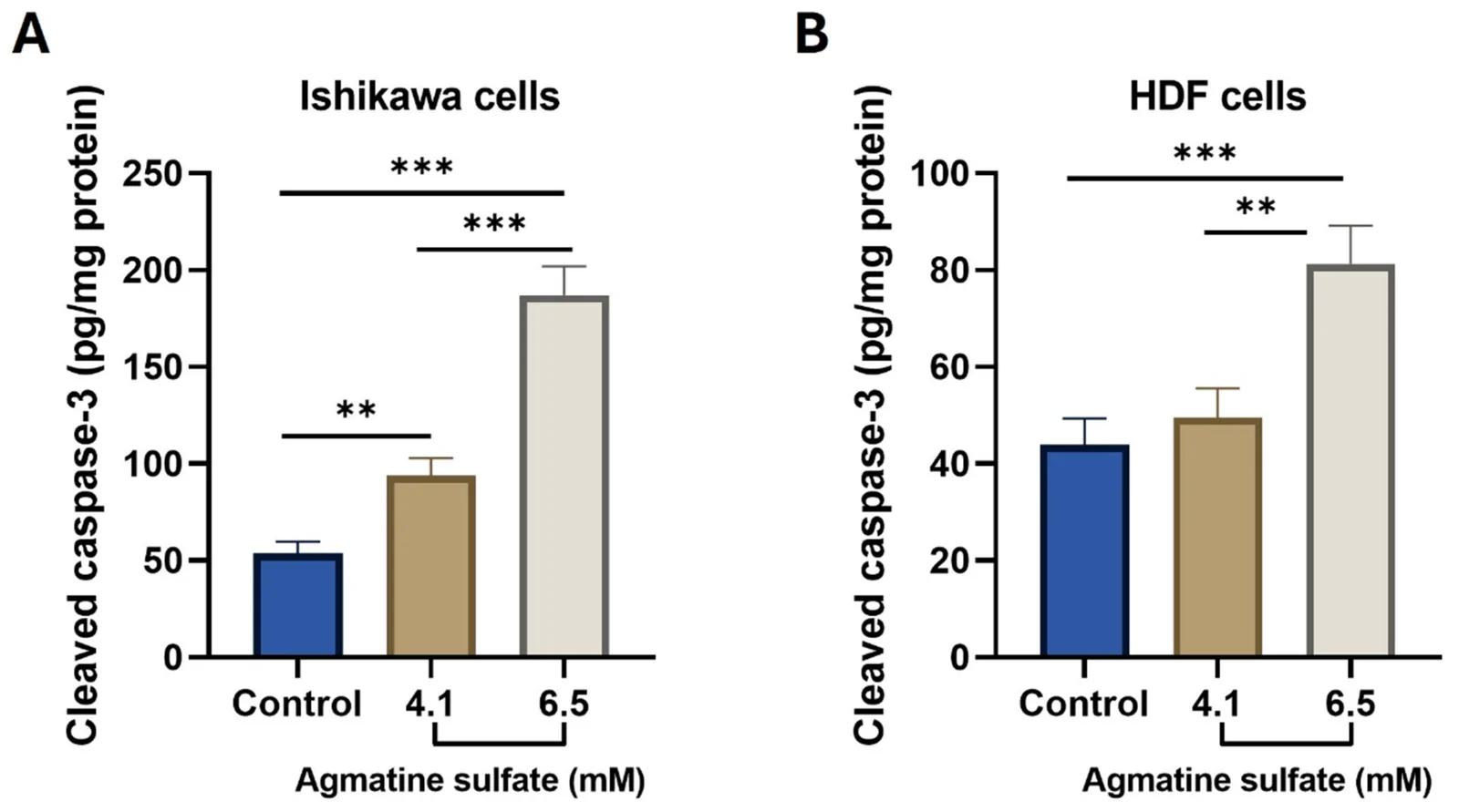
Effects of agmatine sulfate on cleaved caspase-3 levels in Ishikawa and HDF cells. Cleaved caspase-3 levels in (**A**) Ishikawa and (**B**) HDF cells following treatment with 4.1 or 6.5 mM agmatine sulfate for 48 h. Cleaved caspase-3 levels were normalized to total protein content and expressed as pg/mg protein. Data are presented as mean ± SD of three independent experiments (*n* = 3). Statistical significance was determined separately for each cell line using repeated-measures one-way ANOVA followed by Tukey’s multiple comparisons test. Only statistically significant comparisons are indicated. ** *p* < 0.01, *** *p* < 0.001.

Agmatine sulfate increased cleaved caspase-3 levels in Ishikawa cells following 48 h of treatment (Figure 5A). Compared with the untreated control, normalized cleaved caspase-3 levels were significantly increased at both 4.1 mM (*p* = 0.0088) and 6.5 mM (*p* < 0.0001). A further significant increase was observed at 6.5 mM compared with 4.1 mM (*p* < 0.0001).

In HDF cells (Figure 5B), cleaved caspase-3 levels were not significantly altered by treatment with 4.1 mM agmatine sulfate compared with the untreated control (*p* = 0.5692). In contrast, treatment with 6.5 mM significantly increased cleaved caspase-3 levels compared with both the untreated control (*p* = 0.0010) and the 4.1 mM treatment group (*p* = 0.0025). Thus, the increase in cleaved caspase-3 was evident at both concentrations in Ishikawa cells, whereas a significant increase in HDF cells was observed only at 6.5 mM.

### 3.5. Effects of Agmatine Sulfate on p-AKT and p-JNK Immunoreactivity

**Figure 6:**
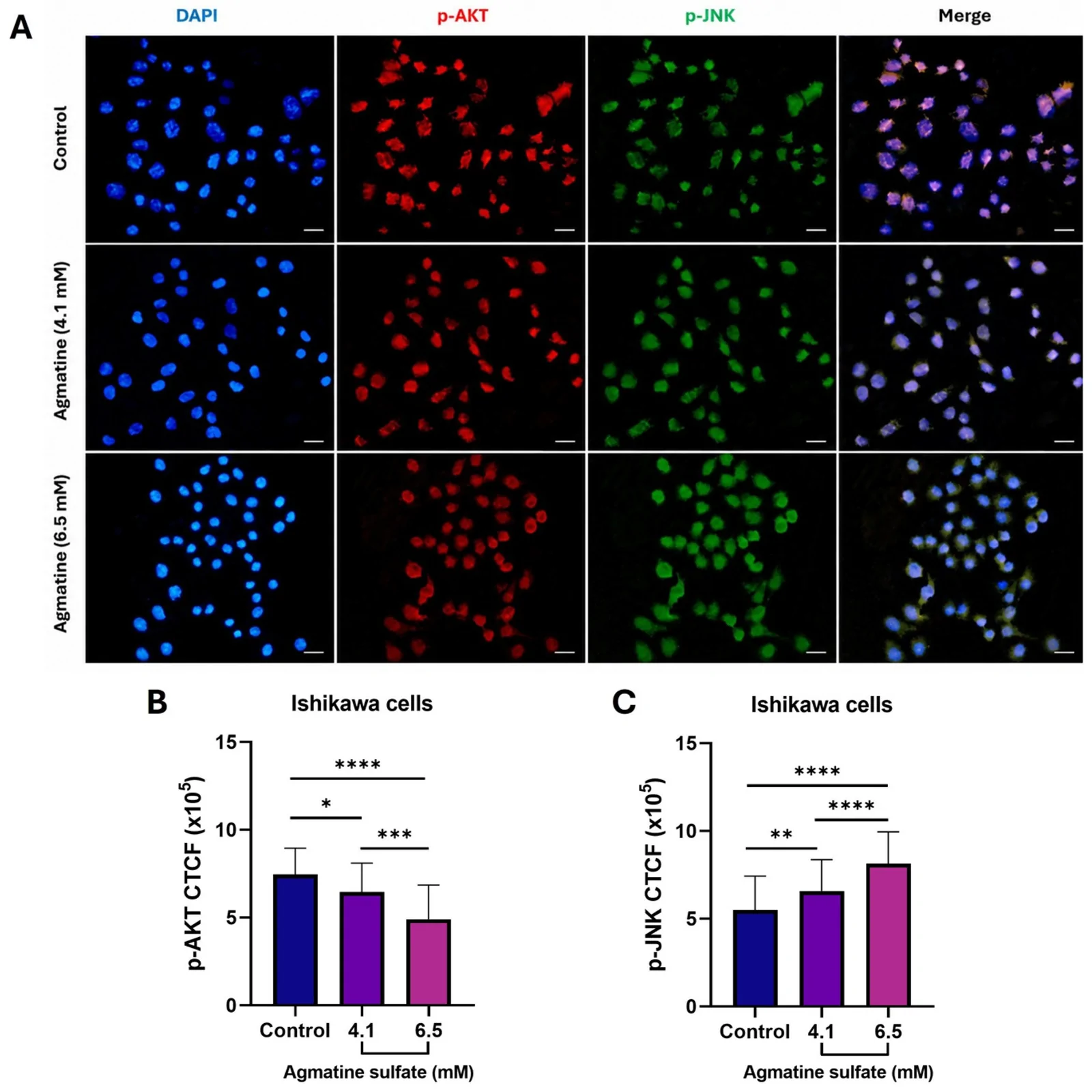
Effects of agmatine sulfate on p-AKT and p-JNK immunoreactivity in Ishikawa cells. (**A**) Representative double immunofluorescence images showing p-AKT (red) and p-JNK (green) immunoreactivity in untreated control cells and cells treated with 4.1 or 6.5 mM agmatine sulfate for 48 h. Nuclei were counterstained with DAPI (blue), and merged images are shown in the right column. Scale bars = 100 μm. (**B**) Quantitative CTCF analysis of p-AKT immunoreactivity and (**C**) p-JNK immunoreactivity. Data are presented as mean ± SD of three independent experiments (*n* = 3 independent biological replicates). Statistical significance was determined by repeated-measures one-way ANOVA followed by Tukey’s multiple comparisons test. Only statistically significant comparisons are indicated. * *p* < 0.05, ** *p* < 0.01, *** *p* < 0.001, **** *p* < 0.0001.

Double immunofluorescence staining revealed changes in p-AKT and p-JNK immunoreactivity following agmatine sulfate treatment in Ishikawa cells (Figure 6A). Quantitative CTCF analysis showed a significant reduction in p-AKT immunoreactivity at 4.1 mM compared with the untreated control (*p* = 0.0267), with a greater reduction at 6.5 mM (*p* < 0.0001). p-AKT immunoreactivity at 6.5 mM was also significantly lower than that at 4.1 mM (*p* = 0.0003) (Figure 6B). Mean p-AKT CTCF values decreased from 7.460 × 10^5^ in the control group to 6.461 × 10^5^ and 4.912 × 10^5^ following treatment with 4.1 and 6.5 mM agmatine sulfate, respectively.

In contrast, p-JNK immunoreactivity increased following agmatine sulfate treatment in Ishikawa cells (Figure 6A,C). Quantitative CTCF analysis showed significant increases at both 4.1 mM (*p* = 0.0052) and 6.5 mM (*p* < 0.0001) compared with the untreated control. p-JNK immunoreactivity at 6.5 mM was also significantly higher than that at 4.1 mM (*p* < 0.0001). Mean p-JNK CTCF values increased from 5.505 × 10^5^ in the control group to 6.577 × 10^5^ and 8.130 × 10^5^ at 4.1 and 6.5 mM, respectively (Figure 6C).

**Figure 7:**
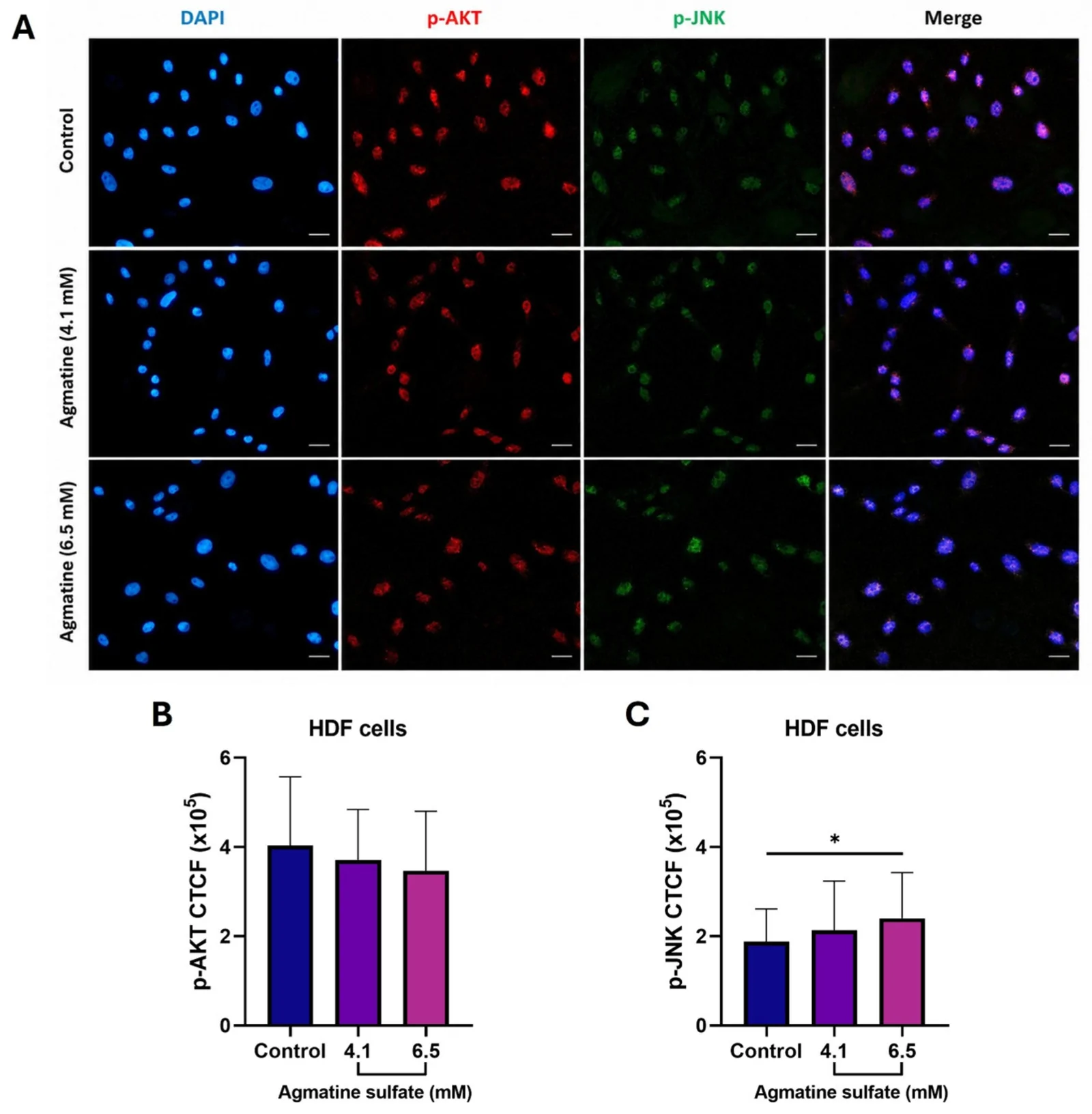
Effects of agmatine sulfate on p-AKT and p-JNK immunoreactivity in HDF cells. (**A**) Representative double immunofluorescence images showing p-AKT (red) and p-JNK (green) immunoreactivity in untreated control cells and cells treated with 4.1 or 6.5 mM agmatine sulfate for 48 h. Nuclei were counterstained with DAPI (blue), and merged images are shown in the right column. Scale bars = 100 μm. (**B**) Quantitative CTCF analysis of p-AKT immunoreactivity and (**C**) p-JNK immunoreactivity. Data are presented as mean ± SD of three independent experiments (*n* = 3 independent biological replicates). Statistical significance was determined by repeated-measures one-way ANOVA followed by Tukey’s multiple comparisons test. Only statistically significant comparisons are indicated. * *p* < 0.05.

In HDF cells, p-AKT immunoreactivity decreased numerically following agmatine sulfate treatment; however, none of the pairwise comparisons reached statistical significance (Figure 7A,B). Mean p-AKT CTCF values were 4.036 × 10^5^ in the control group, 3.711 × 10^5^ at 4.1 mM, and 3.468 × 10^5^ at 6.5 mM. The corresponding adjusted *p* values were 0.5267 for control versus 4.1 mM, 0.1461 for control versus 6.5 mM, and 0.6993 for 4.1 versus 6.5 mM.

For p-JNK, HDF cells showed a numerical increase in immunoreactivity following agmatine sulfate treatment (Figure 7A,C). The difference between the control and 4.1 mM groups was not statistically significant (*p* = 0.4498), whereas p-JNK immunoreactivity was significantly higher at 6.5 mM than in the untreated control (*p* = 0.0434). No significant difference was observed between the 4.1 and 6.5 mM groups (*p* = 0.4430). Mean p-JNK CTCF values were 1.876 × 10^5^, 2.138 × 10^5^, and 2.401 × 10^5^ in the control, 4.1 mM, and 6.5 mM groups, respectively.

Across the two concentrations examined, agmatine sulfate was associated with lower p-AKT and higher p-JNK immunoreactivity in Ishikawa cells, with greater changes observed at 6.5 mM. In HDF cells, p-AKT immunoreactivity was not significantly altered, whereas a significant increase in p-JNK immunoreactivity was observed only at 6.5 mM.

## 4. Discussion

The present exploratory *in vitro* study shows that agmatine sulfate is associated with alterations in metabolic activity, oxidative stress-related parameters, apoptosis-associated responses, and AKT/JNK immunoreactivity in Ishikawa endometrial adenocarcinoma cells. Agmatine sulfate reduced MTT-derived metabolic activity, increased intracellular ROS and MDA levels, and increased cleaved caspase-3 levels in Ishikawa cells. These changes were accompanied by decreased p-AKT and increased p-JNK immunoreactivity. HDF cells exposed to the same concentrations also exhibited changes in oxidative stress markers and cleaved caspase-3 levels, although their signalling responses differed from those observed in Ishikawa cells. These findings indicate that agmatine sulfate exposure is associated with concurrent changes in redox status, apoptosis-associated markers, and stress- and survival-related signalling in Ishikawa cells. However, the present data should be interpreted as evidence of associated cellular response patterns rather than as demonstrating a causal molecular mechanism.

Agmatine sulfate produced concentration- and time-dependent reductions in MTT-derived metabolic activity in both cell lines, with nonlinear regression of the 48-h data yielding lower IC_25_ and IC_50_ values in Ishikawa cells than in HDF cells. These findings are consistent with previous studies reporting growth-inhibitory effects of agmatine in intestinal tumour-cell models. Agmatine has been shown to inhibit the proliferation of several human intestinal tumour cell lines and to exert marked cytostatic effects at millimolar concentrations [16,17]. More recently, concentration-dependent reductions in cell viability have also been reported in Caco-2 colorectal adenocarcinoma cells, with an IC_50_ of 7.3 mM at 72 h [14]. Nevertheless, this difference in IC values between the two cell lines should not be interpreted as evidence of tumour-selective activity because HDF cells are dermal fibroblasts and do not represent a tissue-matched normal endometrial control. It should also be noted that, because 4.1 and 6.5 mM correspond to the Ishikawa-derived IC_25_ and IC_50_, these same absolute concentrations represent a comparatively lower fraction of the HDF-specific IC_25_ and IC_50_ (5.94 and 10.83 mM, respectively); consequently, the weaker or absent responses subsequently observed in HDF cells may partly reflect this lower relative exposure rather than an intrinsic difference in cellular sensitivity to agmatine sulfate. Rather, the concentration-response analysis provided a quantitative basis for selecting 4.1 and 6.5 mM, corresponding approximately to the Ishikawa IC_25_ and IC_50_, for subsequent experiments under identical exposure conditions. Furthermore, because the MTT assay reflects cellular metabolic activity rather than directly measuring cell number or cell death, the observed reductions should not by themselves be equated with cytotoxicity [18]. The oxidative stress and cleaved caspase-3 measurements therefore provide information that the MTT data alone cannot. Agmatine sulfate also increased oxidative stress-related parameters. In Ishikawa cells, intracellular ROS levels increased at both 4.1 and 6.5 mM, with the greatest increase observed at 6.5 mM. MDA levels showed a similar pattern, indicating increased lipid peroxidation in parallel with ROS accumulation [7,9]. The biological effects of ROS depend on cellular context and magnitude. Although moderate ROS levels can participate in proliferative and survival signalling in malignant cells, excessive oxidative stress may exceed cellular antioxidant capacity and contribute to macromolecular damage and activation of cell-death pathways [5,6,9]. The parallel increases in ROS and MDA observed in Ishikawa cells are consistent with an oxidative stress-associated response to agmatine sulfate.

These findings may appear to contrast with previous reports describing antioxidant and cytoprotective effects of agmatine in non-tumour experimental models. In LPS-stimulated macrophages, agmatine was reported to attenuate ROS accumulation through PI3K/AKT-dependent activation of Nrf2/HO-1 signalling [12], whereas in cisplatin-induced ototoxicity models, agmatine exerted cytoprotective effects associated with activation of PI3K/AKT signalling [13]. However, evidence from tumour-cell models suggests that agmatine responses may differ according to cellular context. Antiproliferative and pro-apoptotic effects have been reported in Caco-2 colorectal adenocarcinoma cells [14], while agmatine sulfate has been shown to decrease PI3K and Akt-1 expression in HT1080 human fibrosarcoma cells, in association with reduced MMP-2 expression and activity [15]. Those HT1080 experiments were performed at sub-millimolar concentrations (below 512 μM), well below the millimolar range required to reduce metabolic activity in the present cell lines, so the comparison concerns the direction of the AKT change rather than the exposure level at which it occurs. Thus, the present findings do not necessarily contradict the previously described cytoprotective actions of agmatine; rather, they support the possibility that its redox and signalling effects vary substantially among experimental systems.

The increase in cleaved caspase-3 provides additional evidence of an apoptosis-associated cellular response. In Ishikawa cells, cleaved caspase-3 levels were significantly increased at both 4.1 and 6.5 mM, with a substantially greater increase at the higher concentration. In HDF cells, by contrast, a significant increase was observed only at 6.5 mM. The concomitant increase in ROS, MDA, and cleaved caspase-3 in Ishikawa cells is compatible with an association between increased oxidative stress and activation of apoptotic machinery [6,9]. Nevertheless, the present experimental design does not establish that ROS generation directly caused caspase-3 activation. Establishing such a causal relationship would require additional experiments, such as antioxidant rescue or pharmacological modulation. Accordingly, the current findings are best interpreted as showing parallel oxidative stress- and apoptosis-associated responses following agmatine sulfate exposure.

The p-AKT and p-JNK measurements describe the signalling changes that accompanied these responses. In Ishikawa cells, agmatine sulfate was associated with lower p-AKT immunoreactivity and higher p-JNK immunoreactivity across the two concentrations examined, with greater changes at 6.5 mM. AKT is a major component of survival-associated signalling, whereas JNK is a stress-responsive MAPK whose biological consequences depend strongly on cellular context, stimulus intensity, and duration [3,4,19]. The simultaneous reduction in p-AKT and increase in p-JNK observed here therefore coincided with increased oxidative stress and cleaved caspase-3 levels. This pattern is compatible with a shift from survival-associated towards stress-associated signalling in agmatine-treated Ishikawa cells, consistent with reports showing that oxidative stress can be accompanied by reduced AKT signalling and enhanced JNK activation in cancer cells [20]. The observed changes in p-AKT and p-JNK immunoreactivity should not, however, be interpreted as direct evidence of pathway inhibition or activation, particularly because total AKT and JNK levels were not assessed. The present results therefore support an association between agmatine sulfate exposure and changes in AKT/JNK-related signalling rather than proving that these pathways mediate the observed changes in metabolic activity or apoptosis.

In HDF cells, p-AKT immunoreactivity was not significantly altered at either concentration, whereas p-JNK immunoreactivity was significantly increased only at 6.5 mM. At the same time, ROS levels were significantly increased at both concentrations, whereas MDA and cleaved caspase-3 levels were significantly elevated only at 6.5 mM. These observations indicate that HDF cells also exhibit biological responses to agmatine sulfate and therefore do not support a conclusion of tumour-selective activity. Rather, the differing patterns of biochemical and signalling responses between the two cell types suggest that agmatine-associated effects may depend on cellular context. Because HDF cells are derived from dermal tissue, these differences should be considered descriptive and hypothesis-generating rather than evidence of selectivity for endometrial cancer cells.

Several limitations should be considered when interpreting the present findings. First, the study was performed using a single endometrial cancer cell line, which limits the generalizability of the observations across the molecularly heterogeneous spectrum of endometrial cancer [1]. Second, HDF cells were used as a non-cancerous comparator but are not of endometrial origin; therefore, they cannot serve as a tissue-matched normal control or provide definitive evidence of tumour selectivity. In addition, because HDF cells were exposed to concentrations derived from the Ishikawa concentration-response curve rather than concentrations matched to their own IC_25_/IC_50_ values, the comparatively weaker responses observed in HDF cells cannot be fully dissociated from this lower relative dose; experiments applying HDF-specific IC_25_/IC_50_ concentrations would be required to determine whether cell-type-dependent differences persist under matched relative exposure. Future studies incorporating normal endometrial epithelial or stromal cells together with additional endometrial cancer cell lines would allow cell-type-specific responses to be evaluated properly. Third, p-AKT and p-JNK were assessed by quantitative immunofluorescence, without complementary Western blotting or measurement of total AKT and JNK. Consequently, the findings describe changes in phosphorylated-protein immunoreactivity but do not establish changes in phosphorylation ratios or direct pathway regulation. Fourth, although the parallel increases in ROS, MDA, and cleaved caspase-3 are consistent with an oxidative stress-associated apoptotic response, no antioxidant rescue or pathway-specific inhibition experiments were performed, precluding causal conclusions regarding the relationship between oxidative stress, AKT/JNK signalling, and apoptosis. Fifth, Ishikawa and HDF cells were cultured in their supplier-recommended but different basal media (RPMI-1640 and DMEM, respectively), and medium pH and osmolality were not measured after agmatine sulfate supplementation; although the millimolar concentrations used are expected to produce only modest osmotic shifts, a contribution of medium-related factors to the observed responses cannot be fully excluded. Finally, the downstream analyses were conducted at a single 48-h time point and at two concentrations selected from the Ishikawa concentration-response curve; consequently, while MTT-derived metabolic activity was characterized across a full concentration range, the ROS, MDA, cleaved caspase-3, and p-AKT/p-JNK data describe a trend between these two concentrations rather than a complete concentration-response relationship for these endpoints; additional time-course and targeted experimental studies will be required to define the temporal sequence and biological significance of these responses.

Within these limits, the present study characterizes agmatine sulfate-associated responses in Ishikawa endometrial adenocarcinoma cells across metabolic, redox, apoptotic, and signalling readouts. The combination of concentration-response analysis with measurements of intracellular ROS, MDA, cleaved caspase-3, and p-AKT/p-JNK immunoreactivity shows that reduced MTT-derived metabolic activity is accompanied by increased oxidative stress and apoptosis-associated responses, together with changes in stress- and survival-related signalling. Clarifying how redox changes, AKT/JNK signalling, and cell death relate to one another will require additional endometrial cancer cell lines, tissue-matched non-malignant controls, and experiments designed to test those relationships directly.

## Data Availability

The data supporting the findings of this study are available from the corresponding author upon reasonable request. No new genetic or sequencing data were generated, and therefore no data were deposited in public repositories.

## Abbreviations

ROS, reactive oxygen species; MDA, malondialdehyde; PI3K, phosphatidylinositol 3-kinase; AKT, protein kinase B; p-AKT, phosphorylated AKT; JNK, c-Jun N-terminal kinase; p-JNK, phosphorylated c-Jun N-terminal kinase; CTCF, corrected total cell fluorescence; HDF, human dermal fibroblast; MTT, 3-(4,5-dimethylthiazol-2-yl)-2,5-diphenyltetrazolium bromide; DCFDA, 2′,7′-dichlorofluorescein diacetate; DMSO, dimethyl sulfoxide; TBHP, tert-butyl hydroperoxide; ELISA, enzyme-linked immunosorbent assay; ECACC, European Collection of Authenticated Cell Cultures; ATCC, American Type Culture Collection; PBS, phosphate-buffered saline; BSA, bovine serum albumin; BCA, bicinchoninic acid; DAPI, 4′,6-diamidino-2-phenylindole; ANOVA, analysis of variance; MAPK, mitogen-activated protein kinase; mTOR, mechanistic target of rapamycin; SD, standard deviation; LPS, lipopolysaccharide; MMP-2, matrix metalloproteinase-2.
